# Evaluation of a multifaceted "Resident-as-Teacher" educational intervention to improve morning report

**DOI:** 10.1186/1472-6920-6-20

**Published:** 2006-03-26

**Authors:** Matthew T James, Marcy J Mintz, Kevin McLaughlin

**Affiliations:** 1 Department of Medicine, University of Calgary, Foothills Medical Centre,1403-29^th ^Street NW, Calgary, AB, T2N 2T9, Canada

## Abstract

**Background:**

Resident-led morning report is an integral part of most residency programs and is ranked among the most valuable of educational experiences. The objectives of this study were to evaluate the effect of a resident-as-teacher educational intervention on the educational and teaching experience of morning report.

**Methods:**

All senior internal medicine residents were invited to participate in this study as teaching participants. All internal medicine residents and clerks were invited to participate as audience participants. The educational intervention included reading material, a small group session and feedback after teaching sessions. The educational and teaching experiences were rated prior to and three months after the intervention using questionnaires.

**Results:**

Forty-six audience participants and 18 teaching participants completed the questionnaires. The degree to which morning report met the educational needs of the audience was higher after the educational intervention (effect size, d = 0.26, p = 0.01). The perceptions of the audience were that delivery had improved and that the sessions were less intimidating and more interactive. The perception of the teaching participants was that delivery was less stressful, but this group now reported greater difficulty in engaging the audience and less confidence in their medical knowledge.

**Conclusion:**

Following the educational intervention the audience's perception was that the educational experience had improved although there were mixed results for the teaching experience. When evaluating such interventions it is important to evaluate the impact on both the educational and teaching experiences as results may differ.

## Background

Morning report is an integral part of most medical residency programs across North America. The format is typically a presentation of a recent clinical case and discussion of this case led by one or more residents with attending physicians, residents, and medical students participating in the case discussion. While originally implemented to help oversee the care of patients, the focus of morning report has more recently shifted towards education [1]. The impetus for change has been supported by surveys showing that residents feel education should be the primary purpose of morning report [2]. Indeed a recent survey of resident attitudes found that morning report was ranked as the most valuable educational activity within residency curriculum [3].

The responsibility of organizing and delivering morning report and facilitating the subsequent discussion typically falls on senior residents. This choice would appear to be appropriate as residents report teaching as a valuable and enjoyable part of their training [4,5]. Moreover, the valuable contribution of residents as teachers is becoming increasingly recognized [6-8]. Despite these teaching demands, residents in many programs are given little, if any, formal instruction on how to become effective presenters [9]. A recent review on the subject of teaching residents to teach reports several educational interventions that were found to be of benefit [10]. The results of the fourteen studies reviewed were, however, quite variable and success was often short lasting. An additional criticism of these studies was that the most frequent outcome measure used was residents' self-reported effectiveness, which might not translate into greater effectiveness from the viewpoint of the audience.

To achieve a lasting change in residents' teaching performance the resident must go through the generic stages of behaviour change as described by Prochaska: pre-contemplative; contemplative; preparation; action; and maintenance or relapse [11]. It is unusual for a single intervention to be sufficient to move an individual through all these stages and thus facilitate a lasting behaviour change. Studies that combine interventions have, however, been more successful [12]. Such studies may combine a predisposing intervention (designed to achieve the *preparation *stage of change) with an enabling intervention (designed to achieve the *action *stage of change) and a reinforcing intervention (designed to achieve the *maintenance *stage and prevent the *relapse *stage of change). In this study we designed a multifaceted educational intervention to address the various stages of change and, hopefully, facilitate a lasting improvement in the teaching behaviour of internal medicine residents during the morning report. Our study was also designed to overcome some of the criticisms of previous studies by evaluating several outcome variables that may change as a result of the educational intervention.

The first objective of this study was to evaluate the effect of a multifaceted educational intervention aimed at improving the delivery of morning report on the perceived effectiveness of morning report as an educational tool. The second objective was to identify which aspects of the educational experience, if any, changed as a result of the educational intervention. The third objective was to identify which aspects of the teaching experience, if any, changed as a result of the educational intervention.

## Methods

### Study participants

All University of Calgary senior (second and third year) internal medicine residents were invited to participate in this study as teaching participants. All senior residents had served as preceptors for morning report over a period of at least five months. All senior and junior (first year) residents as well as all clinical clerks attached to the medical teaching unit were invited to participate as audience participants in this study. Written consent was obtained from all participants.

### Educational intervention

#### Predisposing intervention

During the workshop (see below) teaching participants were given educational material containing advice and recommendations on how to improve teaching to small and large groups of learners [13-15].

#### Enabling intervention

Teaching participants attended a three-hour workshop on how to give morning report. The workshop, presented by two teaching faculty members (MM and KM), was held during the usual time of the weekly academic half-day. The workshop was designed to focus on strategies for effective presentation of the morning report. Specifically, instruction was provided in six areas: choosing learning objectives; selecting content; identifying key teaching points; delivering content effectively; engaging the audience; and continuing learning after the teaching session has finished. The workshop began with a brainstorming session on 'what went wrong' and 'what went right' during a teaching session in which participants were asked to identify reasons why teaching sessions that they gave or witnesses succeeded or otherwise. Following this participants were divided into four groups and each group was given a teaching scenario. An example of a scenario is given below:

"17 year-old female presents to ER with fever. Final diagnosis acute bacterial meningitis."

Each area of instruction was introduced by one of the by faculty members following which the participants performed the relevant task for their case, e.g., choosing learning objectives for their scenario. When all tasks were completed each small group presented their outline for delivering their teaching session including techniques for delivery, audience participation and facilitation of further learning. After the small group presentations the faculty described ways of using reflection and feedback to improve teaching. The session ended with a review of the literature on morning report and participants were provided with literature on how to improve teaching to small and large groups of learners (see predisposing intervention).

#### Reinforcing intervention

Over the four months following the workshop, the course preceptors provided feedback to senior resident presenters immediately after each morning report session. These informal, short discussions addressed any questions or concerns raised by the resident or brought to light by the preceding presentation, and were designed to reinforce the elements taught earlier in the workshop.

### Data collection

To achieve the first two objectives of this study (to evaluate the effect of the educational intervention on the perceived effectiveness of morning report as an educational tool and to identify which aspects of the educational experience changed as a result of the educational intervention) a Likert-style questionnaire was given to the audience participants prior to the start of the study and then three months after the workshop. The questionnaire had seven questions (shown in table 1) and participants were asked to rate their degree of agreement with the statements (1 = strongly disagree, 2 = disagree, 3 = neutral, 4 = agree, 5 = strongly agree). To achieve the third objective (to identify which aspects of the teaching experience changed as a result of the educational intervention) a separate questionnaire was given to the teaching participants prior to the start of the study and then three months after the workshop. This was a similar questionnaire to the previous but had only five items (see table 2). The same residents were studied before and after the educational intervention. As the clerkship attachment in internal medicine is only three months in duration the group of clerks studied was, therefore, the group at the end of the bock prior to the educational intervention and the group at the end of the block after the educational intervention.

### Statistical analyses

For each question the mean of individual scores for the degree of agreement on the five-point Likert scale was determined. The mean response for each question before and after the educational intervention was compared using paired t-test. The method described by Cohen was used to calculate effect size [16]. All analyses were two-sided and a p-value of < 0.05 was considered to be statistically significant. Statistical analyses were performed using STATA 7.0 software (Stata Corporation, College Station, Texas).

## Results

Forty-six audience participants completed the initial questionnaire (28 of 31 internal medicine residents and 18 of 18 medical students) and 46 completed the follow-up questionnaire (27 of 31 internal medicine residents and 19 of 19 medical students). Eighteen of 20 senior medicine residents attended the workshop. All of the teaching participants completed both questionnaires.

The degree to which morning report met the educational needs of the audience was significantly higher after the educational intervention although the effect size was relatively small (d = 0.26). Following the educational intervention the perceptions of the audience were that delivery of the morning report teaching sessions had improved and that the sessions were less intimidating and more interactive. The effect sizes for these differences were small to medium. These data are shown in table 1. No differences were observed in the clinical relevance of content selected or in the performance of the attending physicians.

Following the educational intervention the perception of the teaching participants was that delivery of the morning report teaching sessions was less stressful. This difference had a moderate effect size. Interestingly, however, this group now reported greater difficulty in engaging the audience and were now less confident that they had sufficient medical knowledge to present morning report. The effect sizes for these differences were medium to large. These data are shown in table 2. No differences were observed in the degree of difficulty in teaching to the varied levels of knowledge of the audience. The teaching participants also perceived no difference in their presentation skills.

## Discussion

The aim of this study was to create and evaluate an educational intervention designed to improve the degree to which the morning report teaching session met the educational needs of its audience. Based upon the results of the post-intervention questionnaire this aim appears to have been met. Cognizant of the steps involved in behavioural change and the limited utility of single interventions we designed a multifaceted intervention to address each step in the change pathway [11,12]. The nature of the interventions is shown in the figure below. Not shown in this figure is, however, another important component of a successful outcome; willingness to change. The audience of senior medical residents all had previous experience of presenting morning report, some of which was negative, and appeared motivated to change by virtue of 90% attendance at an optional workshop. Thus we believed this group to be already predisposed and receptive to an intervention designed to change behaviour and were already some way along the path to change, e.g., at the contemplative or preparation stage. It is not possible with the design of our study to identify the relative contribution of the individual components to the overall success of the intervention. We did not set out to compare the individual components as it was our belief that they could not be considered as independent, e.g., the success of the reinforcing intervention is dependent upon the success of the preceding interventions.

While the intervention appeared to have positive results from the viewpoint of the audience, there were mixed results on the self perceived challenges and competencies of residents presenting morning report. After the intervention resident teachers scored their competencies in engaging the audience lower. This perception of failure to engage the audience was, however, countered by the audience's perception of the teaching sessions being more interactive i.e., they felt more engaged. The reasons for this discrepancy are unclear but, anecdotally, may relate to the fact that most of the post-session feedback involved ways of making the rounds more interactive. This aspect of the intervention may have changed the resident's perception of their performance towards that of the feedback...;'if they keep telling me about ways of making the rounds interactive then I must be doing a bad job at engaging the audience'. Changing the nature of the feedback could test this hypothesis. After the intervention resident teachers also scored their medical knowledge lower. Once again this was an unexpected result and may have been introduced by simply having a session on morning report such that residents feel more scrutinized. Perhaps a more likely explanation is that by reducing anxiety about content selection and delivery, resident teachers became more aware of potential deficiencies in the knowledge base. Reassuringly, the objective information collected for each year of residency shows that residents in our program actually increase their medical knowledge with time. Irrespective of the explanation for the discrepant results they highlight the importance of evaluating the impact of such interventions from more than one aspect [10].

There are some important limitations to our study. Our sample size, despite high enrolment of available residents and students, was small by virtue of this being a single centre study. Conducting this study in several centres would increase the precision of our results although this might also introduce greater variability in the nature of the delivered intervention. In this study we did not have a control group, a decision made based upon the small sample size. It could be argued, therefore, that the changed perceptions observed occurred merely as a result of time and increased experience in presentation, or were due to some other unforeseen intervention. Reassuringly, there were no format changes made to the morning report sessions and no other educational interventions for residents over the course of this study. Repeating this study and involving a second centre to either serve as a control group or to increase the numbers such that a randomized controlled trial were possible would be one way of addressing this limitation. A third limitation is the fact that the effect sizes for the observed changes were, for the most part, small to medium. It is worth noting however, that the levels of satisfaction with the morning report sessions were high prior to the intervention. Consequently, it should be expected that the effect size will be less when aiming to make something that is already quite good better, compared to trying to improve a weak area of the curriculum.

It is worth noting that the outcome measure for this study was the degree to which the teaching session met the educational needs of the audience. We did not set out to evaluate what these education needs specifically were for our audience as previous surveys of morning report have suggested that the provision of medical knowledge, and development of clinical approaches and critical reasoning are amongst the most important [2,3]. Future examination of this teaching skills intervention could examine which of these individual educational needs are improved upon by the intervention. This in turn my help tailor future teaching interventions to address specific educational needs. It remains to be seen whether an increase in the educational value of a session such as this translates into improved competence of the audience.

## Conclusion

In summary, the results of this study suggest that our multifaceted educational intervention facilitated an improvement in the educational value of the morning report teaching session that persisted for at least three months. As the skills learned in this educational intervention are generic it is also hoped that this intervention may also improve the educational value of other resident teaching sessions. This is important to both the residents, as they are evaluated on their teaching performance and to students as the majority of their clinical teaching is actually provided by residents. Further studies are required to compare different educational interventions and also to consider the impact of these on higher-level end-points, such as resident and student competency.

## Competing interests

The author(s) declare they have no competing interests.

## Authors' contributions

MJM and KM were responsible for the conception of the study. MTJ, MJM, and KM all contributed to design, analysis, and interpretation of data. MTJ was responsible for drafting the original article, and MJM, and KM were responsible of revising it critically for intellectual content. All authors approved the final version to be published.

## Pre-publication history

The pre-publication history for this paper can be accessed here:



## Figures and Tables

**Figure 1:**
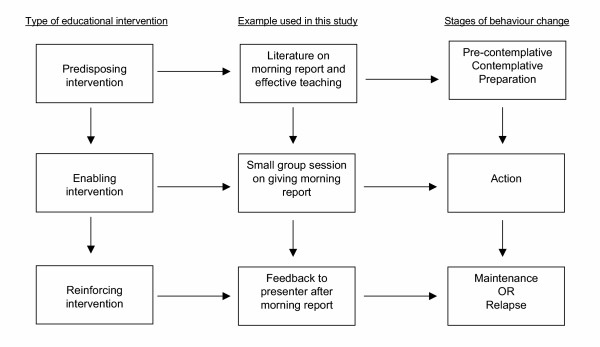
Types of educational interventions designed to change behaviour.

**Table 1:** The effect of the educational intervention on the educational experience

**Question**	Pre-intervention*	Post-intervention*	**Effect size (d)**	**P-value**
The morning report format is an effective format for teaching	4.27 ± 0.75	4.44 ± 0.66	0.24	0.019
Overall the content chosen for morning report was clinically relevant	4.30 ± 0.63	4.39 ± 0.58	0.15	0.16
Overall the delivery of morning report was effective	3.93 ± 0.57	4.17 ± 0.57	0.42	0.0015
As an audience member, the morning report sessions were intimidating	2.58 ± 0.89	2.13 ± 0.76	0.54	<0.0001
Overall the morning report sessions were interactive	4.02 ± 0.58	4.22 ± 0.51	0.37	0.0019
Overall the attending physicians who were at morning report asked appropriate questions	3.91 ± 0.74	3.98 ± 0.66	0.1	0.54
Overall the morning report session met my educational needs	3.96 ± 0.64	4.13 ± 0.69	0.26	0.0098

* mean (± 0.SD) level of agreement with the statement where 1 = strongly disagree, 2 = disagree, 3 = neutral, 4 = 1 = agree, and 5 = strongly agree

**Table 2:** The effect of the educational intervention on the teaching experience.

**Question**	Pre-intervention*	Post-intervention*	**Effect size (d)**	**P-value**
As a presenter, morning report was very stressful	3.00 ± 0.88	2.57 ± 1.02	0.45	0.008
As a presenter, it was easy to engage the audience to participate	2.71 ± 0.91	2.07 ± 0.83	0.74	0.0003
As a presenter, the greatest challenge was to teach at the various levels of the audience	3.93 ± 0.83	3.79 ± 0.58	0.2	0.34
I feel that I have sufficient medical knowledge to present morning report	3.61 ± 0.78	3.22 ± 0.88	0.47	0.015
I feel that I have sufficient presentation skills to present morning report	3.33 ± 0.69	3.39 ± 0.85	0.01	0.58

* mean (± 0.SD) level of agreement with the statement where 1 = strongly disagree, 2 = disagree, 3 = neutral, 4 = 1 = agree, and 5 = strongly agree
